# Transcriptional Activation of Pericentromeric Satellite Repeats and Disruption of Centromeric Clustering upon Proteasome Inhibition

**DOI:** 10.1371/journal.pone.0165873

**Published:** 2016-11-02

**Authors:** Theona Natisvili, Cihangir Yandim, Raquel Silva, Giulia Emanuelli, Felix Krueger, Sathiji Nageshwaran, Richard Festenstein

**Affiliations:** 1 Gene Control Mechanisms and Disease Group, Department of Medicine, Division of Brain Sciences and MRC Clinical Sciences Centre, Imperial College London, Hammersmith Hospital, London, United Kingdom; 2 Bioinformatics Group, Babraham Institute, Cambridge, United Kingdom; Ludwig-Maximilians-Universitaet Muenchen, GERMANY

## Abstract

Heterochromatinisation of pericentromeres, which in mice consist of arrays of major satellite repeats, are important for centromere formation and maintenance of genome stability. The dysregulation of this process has been linked to genomic stress and various cancers. Here we show in mice that the proteasome binds to major satellite repeats and proteasome inhibition by MG132 results in their transcriptional de-repression; this de-repression is independent of cell-cycle perturbation. The transcriptional activation of major satellite repeats upon proteasome inhibition is accompanied by delocalisation of heterochromatin protein 1 alpha (HP1α) from chromocentres, without detectable change in the levels of histone H3K9me3, H3K4me3, H3K36me3 and H3 acetylation on the major satellite repeats. Moreover, inhibition of the proteasome was found to increase the number of chromocentres per cell, reflecting destabilisation of the chromocentre structures. Our findings suggest that the proteasome plays a role in maintaining heterochromatin integrity of pericentromeres.

## Introduction

Packaging of DNA into chromatin plays an important role in transcriptional regulation. Euchromatin is accessible to the transcription machinery whereas heterochromatin is more compact and associated with transcriptional repression [1]. Multiple factors including transcription factors, post-translational histone modifications and DNA methylation are thought to maintain heterochromatin repression [2]. Among them, histone hypoacetylation, histone 3 lysine 9 trimethylation (H3K9me3) and heterochromatin protein 1 (HP1α) were shown to be required for maintenance of heterochromatin [3, 4]. Constitutive heterochromatin predominantly consists of satellite repeats. In mouse cells, pericentromeric and centromeric satellite repeats are the major and minor satellite repeats respectively [5]. Heterochromatinisation of pericentromeric repeats is important for centromere formation and maintenance of genome stability [6]. Low levels of pericentromeric satellite repeat transcription have been detected under various physiological conditions, including cell cycle, senescence, development and differentiation [7–10]. However, aberrant overexpression of pericentromeric satellite repeats has been detected in several pathological conditions, including cellular stress [11–13], cancer [14–17] and some genetic disorders [18–20].

The proteasome is a highly conserved proteolytic complex comprised of the catalytic 20S core particle (CP) capped at one or both ends by the 19s regulatory particle (RP). It regulates protein quality by recognising, unfolding and degrading polyubiquitin tagged, aged, misfolded or damaged proteins [21–23]. Growing evidence, mainly from studies in yeast, suggests that the proteasome is associated with chromatin and regulates transcription [24–30]. Thus, the proteasome regulates the levels and binding of activators as well as recruitment of co-activators at 5’ regulatory regions, thereby controlling transcriptional initiation [26, 31, 32] as well as elongation [27, 33]. It is also thought to enable release of RNA polymerase II (RNAPII) and thereby regulate transcription termination [34]. Moreover, defects of the proteasome subunits in yeast were shown to enhance transcriptional repression of heterochromatin [35]. Additionally, ubiquitin mediated degradation of the Jmj family protein Epe1 was shown to be required for the accurate formation of heterochromatin boundaries [36]. Notably, few studies, mostly in mammalian cells, suggest that the proteasome also regulates transcriptional repression. For example, inhibition of the 20S proteasome resulted in increased levels of RNAPII and the active chromatin mark H3K4me3 at the glucocorticoid responsive gene promoter, where proteasome binding was identified in human cells [37]. Another study proposed that the proteasome blocks nonspecific transcription initiation by preventing formation of the preinitiation complex at cryptic transcription sites [38] and degrades RNAPII or a member of the pre-initiation complex that drives the transcription at these ectopic sites, thereby suppressing transcription. Moreover, a study performed on rat liver showed that proteasome inhibition led to global histone hypomethylation (especially at H3K9 and H3K27 residues) and hyperacetylation [39]. Here we demonstrate that proteasomal activity in mice is also involved in the repression of pericentromeric satellite repeat expression and integrity of pericentromeric clusters.

## Results and Discussion

### Binding of the 20S proteasome at major satellite repeats

**Fig 1:**
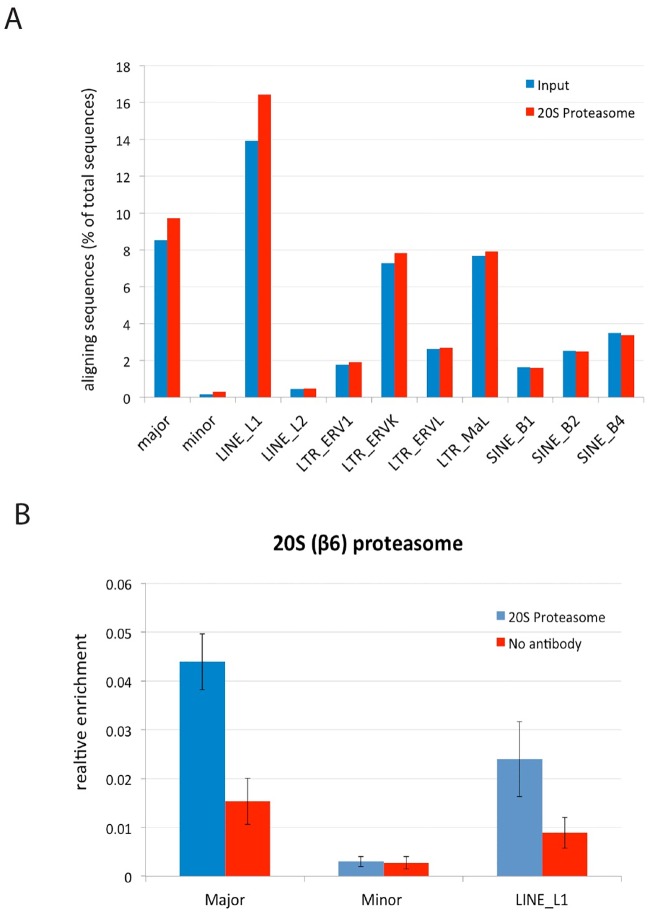
Binding of the 20S proteasome particle at major satellite repeats. (A) ChIP-seq data analysis obtained by ChIP against FLAG-tagged β1 subunit (PSMB1) of the 20S proteasome particle in the mouse 3T3-L1 cell line. The enrichment was greater at major and minor satellites as well as LINE_L1 elements but not other classes of DNA repetitive elements. (B) ChIP-qPCR analysis using antibody against β6 subunit of the 20S proteasome and no antibody control performed on the mouse NIH3T3 cell line. Enrichment level is shown relative to input after subtraction of background. Error bars = SEM of 3 biological replicate.

Several studies have shown the presence of the proteasome in eukaryotic nuclei [40–44] and its recruitment to chromatin including centromeres [45], telomeres [46] and sites of cryptic transcriptional initiation [38]. To investigate whether the proteasome might participate in transcriptional silencing of heterochromatin, proteasome binding at pericentromeric and several other endogenous repeats was analysed using ChIP-seq data previously obtained in mouse 3T3-L1 cells [47]. The results indicated a ~1.2 fold enrichment of the proteasome at pericentromeric major satellite repeats and LINE L1 elements and ~1.9 fold enrichment at centromeric minor satellite repeats, compared to input, whereas all other elements showed no signal above input (Fig 1A). To replicate this qualitative observation, ChIP was performed in another mouse NIH3T3 cell line using an antibody against the 20S proteasome which confirmed binding of the 20S proteasome to major satellite repeats as well as to LINE L1 elements (Fig 1B). The signal from minor satellite was relatively low and close to background, as was the case for the ChIP-seq data, making it difficult to be precise about the extent of enrichment in this location.

### Transcriptional activation of major satellite repeats expression upon proteasome inhibition

**Fig 2:**
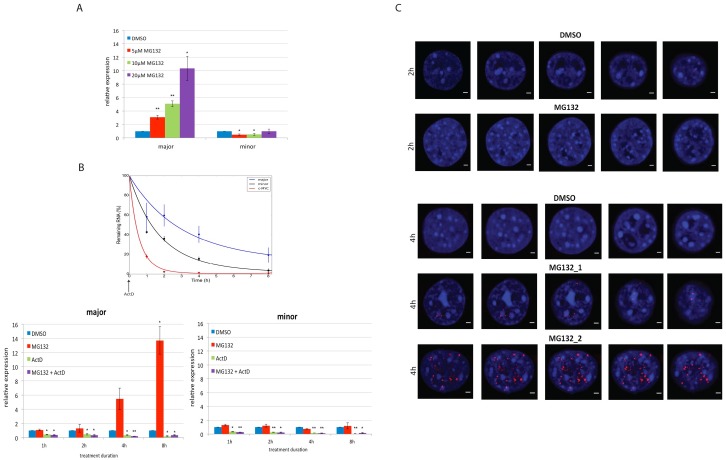
Proteasome inhibition results in upregulation of major satellite repeat expression which is dependent on transcription. **(A) Dose-dependent upregulation of major satellite repeat expression upon proteasome inhibition** NIH3T3 cells were treated with 5μM, 10μM and 20μM MG132 for 8h and transcript levels of major satellite and minor satellite were measured by q-RT-PCR. The relative expression was normalised against spiked RNA and shows fold change relative to DMSO (vehicle). Error bars = SEM of 6 biological replicates. **(B) Transcription-dependent upregulation of major satellite repeat expression upon proteasome inhibition.** Kinetics of the effect of the proteasome inhibition and/or RNAPII inhibition on the expression of major satellite repeats. The right graph indicates the efficiency of the transcriptional inhibition measured by decay of the *c-MYC* and major transcripts. The exponential decay curve was obtained using best fitted nonlinear regression model. NIH3T3 cells were treated with either 20μM MG132, 20μg/ml ActD or both 20μg/ml ActD and 20μM MG132 for 1h, 2h, 4h and 8h followed by RNA extraction and q-RT-PCR. The relative expression was normalised against spiked RNA or Gapdh and shows fold change relative to DMSO. Error bars = SEM of 3 biological replicates. **(C) RNA FISH imaging of major satellite repeat transcription upon proteasome inhibition** NIH3T3 cells were treated with 20μM M132 for 2h and 4h followed by RNA-FISH analysis using major satellite probe. Representative mid-zone confocal z-sections images for DAPI (blue), major (red) and merge are shown for 2h and 4h with DMSO and MG132 treatment. Scale bar 2μm. * p<0.05, ** p<0.001 (Student’s ‘t’ test).

To assess the role of the proteasome at major satellite repeats, pericentromeric transcription was assessed in NIH3T3 cells treated with a widely used and specific proteasome inhibitor MG132. A dose-dependent increase in transcript level of major satellite repeat was observed upon MG132 treatment, reaching 10-fold in treated cells when compared to dimethyl sulfoxide (DMSO—vehicle) (Fig 2A), whereas no effect was seen on the minor satellite expression (Fig 2A). The increase in the major satellite transcript levels could be due to an increase in transcription or to inhibition of major satellite transcript degradation. To assess whether transcription was required for the increase in major satellite repeats transcripts, cells were treated with the transcriptional inhibitor Actinomycin D (ActD) concomitant with MG132. Transcription was effectively inhibited (Fig 2B) and the increase of the major satellite repeat transcription upon proteasome inhibition was blocked in cells treated concomitantly with both MG132 and ActD (Fig 2B), indicating that their upregulation in response to MG132 was indeed transcription dependent. This was further validated at the single cell level using RNA fluorescent in situ hybridization (RNA FISH) targeting major satellite transcripts. Proteasome inhibition led to an increase in the major satellite repeat expression in MG132 treated cells for 4h (Fig 2C). Major satellite signal was located surrounding or within a proportion of DAPI-dense heterochromatin regions showing the characteristic morphology of chromocentres [48]. Signal was also identified in areas of the nucleus lacking chromocentres suggesting either partial transcription from intergenic major satellite DNA sequences or migration of the transcript away from the chromocentres (Fig 2C). Thus, the RNA-FISH analysis is consistent with the previous observation that major satellite repeat transcription was upregulated upon proteasome inhibition (Fig 2A and 2B).

### Upregulation of major satellite repeats upon proteasome inhibition occurs independently of cell cycle perturbation

**Fig 3:**
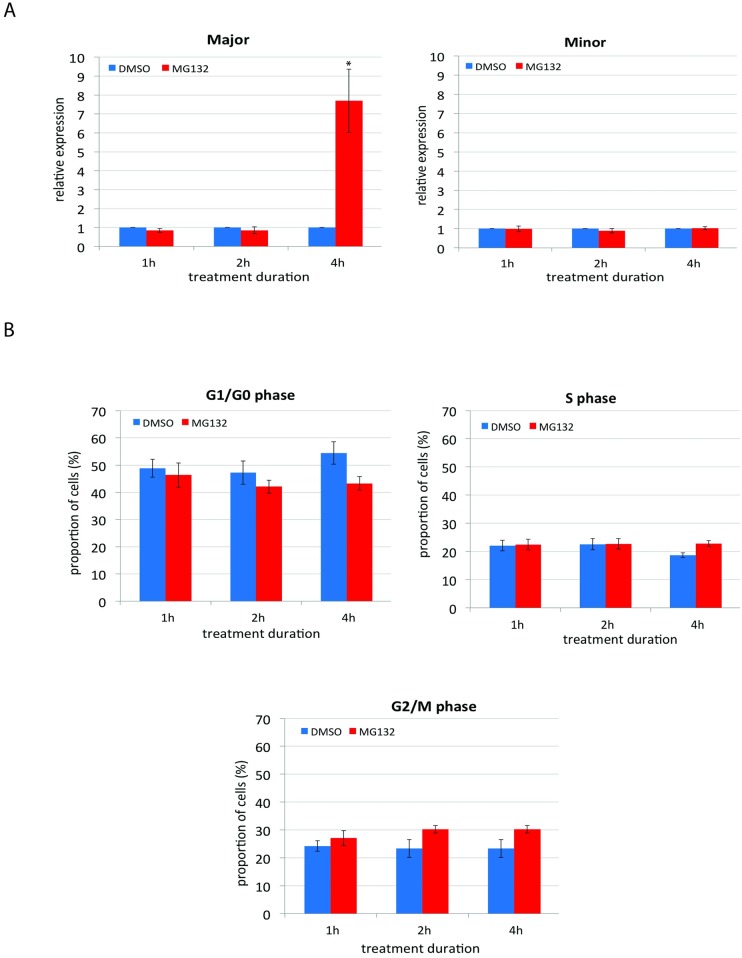
Proteasome inhibition upregulates the major satellite transcription before any significant effect on the cell cycle distribution. **(A) Kinetics of expression of major and minor satellite repeats upon proteasome inhibition.** NIH3T3 cells were treated with 20μM MG132 for 1h, 2h and 4h followed by RNA extraction and q-RT-PCR. The relative expression was normalised against spike and shows fold change relative DMSO. Error bars = SEM of at least 10 biological replicates. Proteasome inhibition upregulated the major satellite repeat expression at 4h of treatment, while minor satellite remained unaffected. **(B) Treatment of cells with proteasome inhibitor MG132 for 4h does not significantly alter the distribution of the cell cycle profile**. NIH3T3 cells were treated with DMSO or 20μM MG132 for 1h, 2h and 4h followed by PI staining and FACS analysis. Proportion (%) of acquired cells in G1/G0, S and G2/M phases are shown for treated and untreated cells. *p<0.05 (Student’s ‘t’ test).

Considering that (i) proteasome inhibition induces cell-cycle arrest in various cell types [49–52] and (ii) cell cycle was shown to regulate the transcription of both pericentromeric and centromeric satellite repeats [7, 53], it was necessary to determine whether the transcriptional activation of major satellite repeats was due to cell cycle skewing. To confirm the cell-cycle dependent transcription of major and minor satellite repeats, counterflow centrifugal elutriation [54] was performed for cell synchronization. Elutriation offers the advantage of selection of cells in different stages of cell cycle and, unlike chemical agents, does not affect the metabolism of cells [54]. The expression of major and minor satellite repeats peaked in G1 and G2/M phases of the cell cycle respectively (S1 Fig), consistent with previous published studies (Ferri et al., 2009; Lu and Gilbert, 2007). We next investigated the effect of proteasome inhibition on (i) the kinetics of transcriptional activation of major and minor satellite repeats and (ii) the cell cycle. Interestingly, the kinetic analysis showed an upregulation of the major satellite repeat expression already at 4h after MG132 treatment (Fig 3A), the time point where no significant effect was seen on the cell cycle profile (Fig 3B). As previously, minor satellite repeat expression was unaffected (Fig 3A). Taken together, these results suggest that upregulation of major satellite repeat expression upon proteasome inhibition is unlikely to be a consequence of cell cycle skewing because the upregulation of the major satellite repeat expression occurred without any significant effects on the cell cycle distribution.

### The effect of proteasome inhibition on chromatin structure

**Fig 4:**
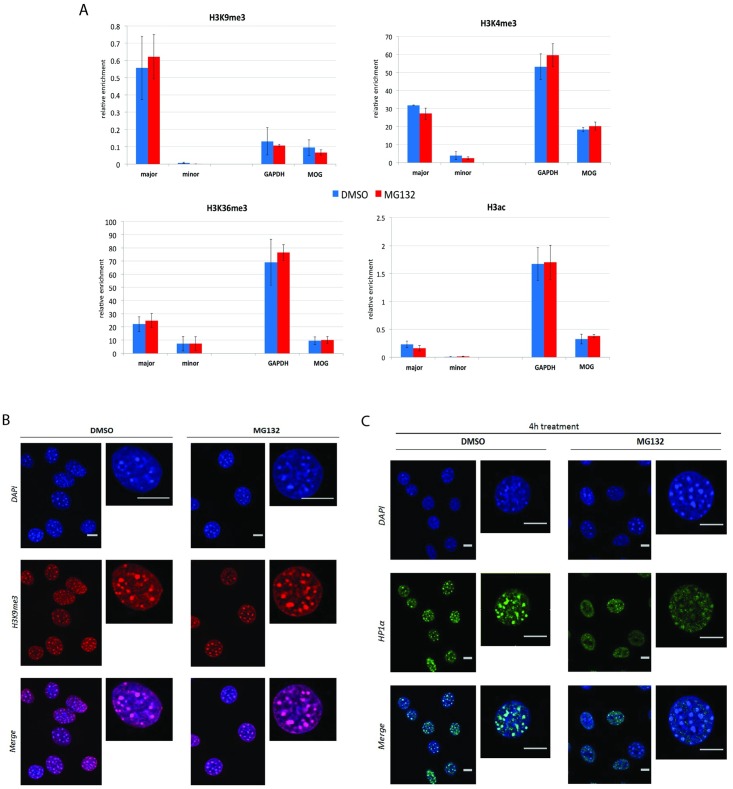
HP1α delocalises from chromocentres upon proteasome inhibition whereas the histone modifications remain unaffected. **(A) Proteasome inhibition does not affect canonical histone modifications on major and minor satellite repeats.** NIH3T3 cells were treated with 20μM MG132 for 4h followed by ChIP–qPCR analysis using antibodies against repressive mark H3K9me3 and activating marks H3K4me3, H3K36me3, and H3ac. Data is shown as relative enrichment to H3 with background subtraction. The y-axis scale was adjusted depending on the signal obtained with different antibodies. Error bars = SEM of 3 biological replicates. **(B) Proteasome inhibition does not affect H3K9me3.** NIH3T3 cells were treated with 20μM MG132 for 4h and immunolabeled with H3K9me3 antibody (red) and stained with DAPI (blue). Scale bar 10μm. **(C) Delocalisation of HP1α from chromocentres upon proteasome inhibition.** NIH3T3 cells were treated with 20μM MG132 for 4h and immunolabeled with HP1α antibody (green) and co-stained with DAPI (blue). Scale bar 10μm.

To assess whether the major satellite repeat upregulation was accompanied by an alteration in the chromatin, histone modifications including H3 acetylation, H3K9me3, H3K4me3 and H3K36me3 were analysed by ChIP-qPCR after treatment of NIH3T3 cells with MG132. H3K9me3, which is considered a “hallmark” of heterochromatin [55] was found to be unaffected by MG132 treatment (Fig 4A). This was further confirmed by immunofluorescence (IF) (Fig 4B). Similar to H3K9me3, the levels of H3 acetylation, H3K4me3 (which is normally associated with promoters of actively transcribed genes [56, 57]) and H3K36me3 (which is normally associated with elongating RNAPII [58, 59]) were found to be similar between cells treated with MG132 and untreated (DMSO) cells (Fig 4A). Thus, the ChIP-qPCR results suggest that proteasome inhibition does not have any net effect on these histone modifications at the major satellite repeat locus. The lack of effect might seem surprising given that proteasome inhibition led to significantly increased transcription of the repeats. This may relate to the proportion of repeat sequences that have been activated by inhibiting the proteasome. The RNA FISH experiment indicates that the number of repeats or heterochromatic clusters that are transcriptionally activated might be a small proportion of the total and therefore any chromatin change would be diluted by the majority of DNA sequences which are not expressing the transcript. This lack of effect on H3K9me3 has been observed in several previous studies (Mosch et al., 2011; Zhu et al., 2011 and see discussion below) where major satellite repeats were found to be upregulated to a similar degree. In contrast, in Pax3/Pax9 deficient iMEFs [60] there was a more significant increase in major satellite transcription and a clear loss of H3K9me3. Lastly, Zhang et al, have similarly reported that transcriptional activation of silent heterochromatin in yeast can occur without any significant changes in the histone modifications [61]. Heterochromatin at pericentromeres is not only regulated by histone modification but also by structural proteins such as HP1α which are involved in chromatin condensation and maintenance of stable heterochromatin [62, 63]. Therefore, the effect of proteasome inhibition on the localisation and distribution of HP1α was evaluated by IF after treatment of NIH3T3 cells with proteasome inhibitor. As expected, in the untreated cells (DMSO), HP1α was concentrated in the DAPI dense stained regions, confirming its localisation to pericentromeric heterochromatin. Interestingly, MG132 treatment resulted in a dispersed distribution of the HP1α protein throughout the nucleus, (Fig 4C and S2A Fig) however, total HP1α levels remained similar between treated and untreated cells (S2B Fig). Thus, proteasome inhibition led to displacement of HP1α from chromocentres, without visible changes in the DAPI dense staining domains. Whether HP1α is displaced specifically from pericentromeric heterochromatin (or from other repressed genomic loci that are associated with chromocentres) remains to be shown. Furthermore this observation is consistent with previous studies where loss of HP1 from pericentromeric heterochromatin was not sufficient to disrupt the DAPI dense stained regions or H3K9me3 [64, 65]. It is also in line with previous reports [64, 66], where transcriptional activation of pericentromeric repeats was accompanied by either partial or full displacement of HP1 from pericentromeres without any changes in H3K9me3 levels, the latter of which serves as a platform for HP1 recruitment to chromatin [67, 68]. Also, HP1α/β double knockout in MEF cells led to upregulation of major satellite repeat expression without affecting the localization of H3K9me3 [64]. Another example comes from BRCA1-deficient cells, where a significant reduction in the number of HP1 positive foci and loss of ubiquitylation of histone H2A (H2Aub) was reported to result in activation of major and minor satellite repeat transcription [66]. Lastly, mutation of variant H3.3 at an early stage in development resulted in increased accumulation of major satellite repeat transcripts which was accompanied by displacement of HP1 from chromocentres [69]. Therefore, it is tempting to speculate that dissociation of HP1α, as seen here, could be sufficient for the remodelling of heterochromatin rendering it accessible to the transcriptional machinery.

**Fig 5:**
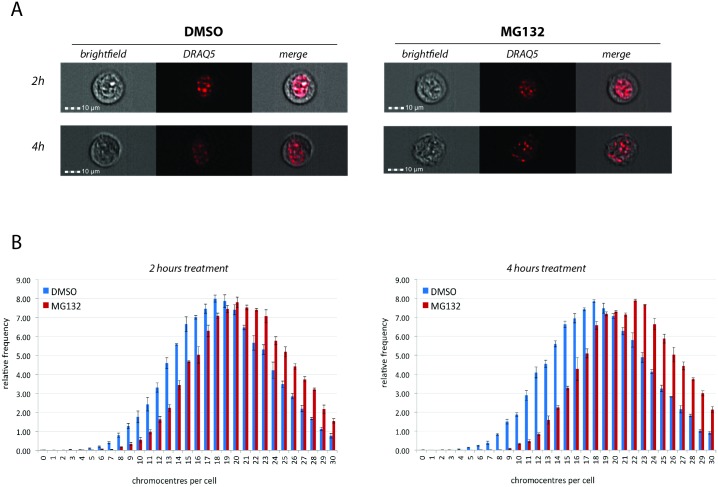
Proteasome inhibition increases the number of chromocentres per cell. NIH3T3 cells were treated with DMSO (vehicle) or 20μM MG132 for 2h and 4h followed by DRAQ5 staining and imaging using ImageStreamX. **(A)** Representative images from brightfield and DRAQ5 wavelengths for each time point of the treatment. DRAQ5 identified chromocentres. **(B)** Comparison of the distribution of the number of chromocentres per cell upon treatment with MG132 and DMSO for each time point (Chi-squared P<0.0001 at both 2 and 4h). Error bars = SEM of 3 biological replicates.

The immunofluorescence imaging of chromocentres (visualised by DAPI) after treatment with proteasome inhibitor did not reveal any obvious structural changes (Fig 4C). Considering that (i) major satellite repeats reside at these regions and (ii) a previous study suggested that upregulation of major satellite repeat expression was accompanied by a marked decrease in the number of chromocentres [66], here the number of chromocentres was analysed in NIH3T3 cells after proteasome inhibition. To acquire cells in a high-throughput manner, an imaging flow cytometer (ImageStream X) was used. Cells were treated with either DMSO or MG132, followed by staining with DRAQ5, which is less toxic for living cells compared to DAPI [70]. Treatment of cells with MG132 resulted in a shift of the distribution of the number of chromocentres per cell towards the right (Fig 5), which increased with time. It is possible that the increased number of chromocentres per cell after proteasome inhibition might result from eviction of HP1α and destabilisation of chromocentre structure, which in turn could reduce the compaction of heterochromatin and increase accessibility of the transcriptional machinery to the underlying DNA sequences.

## Conclusion

The molecular link between the dysregulation of satellite transcription and genomic stress (Zhu et al., 2011) as well as cancer (Eymery et al., 2009) could open a new avenue for understanding cancer evolution and the design of new therapeutic approaches. Here we showed the binding of proteasome to major satellite repeats and their dysregulation upon proteasome inhibition by MG132. As the proteasome participates in a large number of cellular pathways and controls the steady-state level of many proteins, it is difficult to distinguish between direct involvement of the proteasome on the transcription of the pericentromeric repeat and indirect effects mediated by a protein whose level is controlled by proteasome activity. Several studies have shown that the proteasome degrades stalled RNAPII during transcription-coupled repair [71, 72] and a similar mechanism could potentially operate at major satellite repeats. At pericentromeric repeats the proteasome might function to degrade RNAPII and thereby prevent their expression. Additionally, accumulation of misfolded proteins due to proteasome inhibition is known to trigger cell stress responses [73–80]. Therefore, a component from the cell stress pathway might also be responsible for the de-repression of the major satellite repeat. For example, HSF1 was previously shown to activate the transcription of pericentromeric Satellite II and III repeats in human cells upon heat shock in order to form nuclear stress bodies [13, 81, 82]; this effect was reported to be human-specific (Valgardsdottir et al., 200**8**), but similar factors may play a role in other mammals. It is interesting that the eviction of HP1 and disaggregation of chromocentres shown here precedes the detection of major satellite transcripts suggesting that the proteasome is required for integrity of heterochromatin.

## Materials and Methods

### Cell line

NIH3T3 cell line was maintained in Dulbecco’s modified Eagle’s medium (DMEM) with L-glutamine (PAA laboratories GmbH) supplemented with Foetal bovine serum (Sigma) (10% v/v), Penicillin/Streptomycin (GIBCO^®^) (1% v/v) and GlutamixTM-I 100x (GIBCO^®^) (1% v/v) at 37°C in 5% CO_2._ Cells were treated with MG132 (Sigma) which was dissolved in DMSO; ActD (Sigma) which was dissolved in DMSO.

### Quantitative Reverse Transcription Coupled to PCR

After RNA isolation using TRIZOL^®^ Reagent (Invitrogen) and genomic DNA digestion with a DNA free kit (Ambion), cDNA was synthesized using ThermoScript kit (Invitrogen) and random hexamers following the manufacturer’s instructions. The quantitative PCR was performed using the SYBR^®^ Green Jumpstart^™^ Taq ReadyMix^™^ (Sigma-Aldrich^®^) or SensiMix^™^ (Bioline) in a Chromo4 DNA engine (MRJ) with Opticon Monitor 3 (BioRad) software. A list of primers used throughout this study is shown in S1 Table.

### Flow Cytometric Analysis

Cells were fixed in ice cold 70% ethanol by incubation overnight at -20°C. Next day, the fixed cells were washed three time in PBS and treated with 0.2μg/μl RNAse A (Sigma-Aldrich) in PBS for 20min at 37°C. Cells were washed again in PBS and cellular DNA was stained with 50mg/ml propidium iodide (Millipore) diluted in PBS. Stained cells were quantified by FACS (BD LSRII) and gated according to single-cell population and to DNA content representative of the cell cycle.

### Cell cycle separation by counterflow centrifugal elutriation

Cell cycle separation was conducted using an elutriation system with JE-5.0 elutriator rotor (Beckmann Coulter Inc) equipped with Avanti J-26 XP centrifuge (Beckmann Coulter Inc) and a pump. 2X10^8 cells were harvested and washed twice with Elutriation buffer (3.4mM EDTA and 1% FBS in 1X PBS). In order to obtain a single cell suspension the cell pellet was resuspended in 40ml Elutriation buffer and passed twice through an 18-gauge needle (25G) syringe. Next, the sample was loaded into the pre-assembled elutriation chamber. Throughout the elutriation process the centrifuge was maintained at a constant speed of 1700rpm at 4°C. To obtain elutriation fractions, the flow rate of the elutriation buffer was increased from 8ml/min to 20ml/min by 1ml/min increments. Consecutively 200ml effluent volumes were collected from the centrifuge for each flow rate and cells were pelleted by centrifugation. To assess the quality of synchronization in each elutriated fractions, cells were stained PI followed by FACS analysis. Based on the cell cycle profile similarity, elutriation fractions were further grouped and categorised into 3 different fractions.

### Chromatin immunoprecipitation

ChIP experiments were performed as described previously [83], with few modifications. Briefly, cells were fixed in 1% formaldehyde for 10 min at 37°C. Crosslinking was quenched by adding glycine to a final concentration of 125mM for 5min at room temperature. Cell monolayers were incubated in cold swelling buffer (25 mM HEPES pH 7.9, 1.5 mM MgCl_2_, 10mM KCl, 2.5% NP-40. Adjust pH to 7.9. Add freshly 0.5μl P8430 Protease inhibitor cocktail (Sigma) and 5μl 0.1M PMSF in isopropanal per 1 ml of Swelling buffer) followed by scraping and lysing using a Dounce homogenizer. The nuclear pellet was resuspended in cold sonication buffer (50 mM HEPES pH 7.9, 140 mM NaCl, 1mM EDTA, 1% Triton X-100, 0.1% Na-deoxycholate, 0.1% SDS. Adjust pH to 7.9. Add freshly 0.5μl P8430 Protease inhibitor cocktail (Sigma) and 5μl 0.1M PMSF in isopropanol per 1 ml of Sonication buffer) and sonicated using Biorupter (Diagenode) with high energy. Sheared chromatin was centrifuged twice at 15000g for 15min and the supernatant was used for the immunoprecipitation. Immunoprecipitation was performed by initially sonicating (low energy) the chromatin with antibody or no antibody (negative control) using Biorupter (Diagenode) following an overnight incubation with Dynabeads Protein G (Invitrogen) on a rotating wheel at 4°C. Next day, protein G Dynabeads bound to antibody–chromatin complexed was washed and DNA was purified with iPure Kit (Diagenode) following the manufacturer’s instructions. Antibodies used for ChIP were: H3 (Abcam, Ab1791), H3K9me3 (Millipore, 17–625) H3ac (Abcam, Ab47915), H3K4me3 (Abcam, Ab8580), H3K36me3 (Abcam, Ab9050), 20S β6 proteasome (Enzo Life, BML-PW9000).

For the ChiP-seq analysis, the repeat element annotations for the mouse GRCm38 genome build were downloaded from the RepeatMasker track from the UCSC genome browser web site. GSM841627 and GSM1095381 sequencing reads (Catic et al., 2013) were aligned to repeat genomes of several different repeat families using Bowtie 2 (v2.2.6; default parameters), and hits were counted whereby multiple matches of a sequence are possible. The repeat genomes were constructed by concatenating the sequences of each instance of the repeat into a single repeat genome for that repeat family where individual repeats were separated by NNNNN to prevent sequences from mapping over instance boundaries.

The minor satellite consensus sequence:

TTGTAGAACAGTGTATATCAATGAGTTACAATGAGAAACATGGAAAATGATAAAAACCACACTGTAGAACATATTAGATGAGTGAGTTACACTGAAAAACACATTCGTTGGAAACGGGAT, was added manually to the Minor Satellite repeat genome.

### RNA-FISH

RNA *in situ* hybridization (RNA-FISH) was performed using ViewRNA^™^ ISH Cell Assay kit (Affymetrix, eBioscience) following the manufacturer’s instructions. The images were acquired with a 63X oil-immersion lens using a Leica Microsystems SP5 confocal microscope. Analysis of images was performed by Fiji Image J software. RNA probe: Major satellite RNA probe set was designed and produced by eBioscience (Affymetrix) probe developers. This probe was *V00846* —Mouse Satellite DNA sequence—type 1.

### Immunofluorescence

Cells were fixed with 4% PFA diluted in PBS containing 0.1% (v/v) Triton X100 for 15 min at room temperature followed by three washes with PBS and permeabilisation with 0.5% (v/v) Triton X100 diluted in PBS for 30min at room temperature. Cells were washed again three times in PBS and incubated in 20mM glycine dissolved in PBS for 30min at room temperature. Subsequently, cells were blocked for 1h with PBS+ (1%BSA, 0.1% Casein, 0.02% Fish Skin Gelatin in 1X PBS. pH7.8–8) and incubated with primary antibody appropriately diluted in PBS+ in a dark humidified chamber overnight at 4°C. Cells were washed again three times with PBS and incubated in fluorochrome-conjugated (Alexa488 or Alexa568) secondary antibody diluted to the required concentration in PBS for 1h at RT in a dark humidified chamber. Cells were further washed nine times with PBS and incubated for 15min with DAPI diluted in 1:1000 in PBS at room temperature before mounting them using Vectashield mounting medium (Vector Laboratories). Images were acquired with Leica Microsystems SP5 confocal microscope. Analysis of images was performed by Fiji Image J software. Antibodies used for immunofluorescence were: HP1α (Millipore, 05689), H3K9me3 (Millipore, 17–625)

### ImagestreamX

0.5 million NIH3T3 cells were pelleted and washed twice in cold PBS++ (PBS containing 1mM EDTA and 0.02% (w/v) Sodium azide) and resuspended in 100μl of 1μM DRAQ5 dissolved in PBS++. 20000 events were collected at 40X magnification in bright field and the 658nm laser wavelength with ImageStreamX (Amnis, Seattle, Washington). Raw data was quantitated using the associated Image analysis software (IDEAS Amnis). After single cell and DRAQ5 fluorescence gating, the number of chromosome clusters per cell was determined by computing the intensity of localized Draq5 bright spots within the image that were greater than 2.75 pixels in radius.

### Western blot

Cells were lysed using RIPA buffer (50mM Tris pH 8.0, 150mM NaCl, 0.5% Na-deoxycholate, 1% NP-40, 0.1% SDS. Freshly were added 0.5μl P8430 Protease inhibitor cocktail (Sigma) and 5μl 0.1M PMSF in isopropanol per 1 ml of RIFA buffer) and cell extract was cleared by centrifugation at 12 000g for 15min. The supernatant was collected and the protein concentration was determined by Bradford dye colorimetric assay (Bio-rad), following the manufacturer’s instructions. For protein denaturation, lysate samples with specific amounts of protein were mixed with 6X Laemmli buffer (Alfa Aesar) followed by incubation at 100°C for 10min. 5μg of protein was loaded on a SDS-PAGE gel and when electrophoresis was completed, the proteins were electro-transferred from SDS-PAGE gel to pre-washed PVDF membrane (GE Healthcare) in the presence of Transfer buffer (25mM Tris pH 8.3, 190mM glycine. Add freshly 20% (v/v) methanol). The membrane was blocked in blocking buffer (0.1% (v/v) Tween 20, 5% non-fat milk in PBS) for 1h at room temperature followed by incubation with the primary antibody in blocking buffer on a rocking platform overnight at 4°C. After washing the membrane in PBS supplemented with 1% Tween, the membrane was incubated with secondary antibody conjugated with Horse Radish Peroxidase (HRP) in blocking buffer for 1h at room temperature. The presence of HRP on the membrane was then detected using ECL Plus Western Blotting Detection Reagents (GE Healthcare) using the manufacturer’s instructions.

Primary antibodies used were: HP1α (Millipore, MAB3446) was used at 1:500; α-tubulin (Sigma, T5168) at 1:10000. Secondary HRP-goat anti mouse antibody (Life Technologies, G21040) was used at 1:20000.

## Supporting Information

S1 Fig Transcription of major satellite peaks in G1 phase and minor satellite in G2/M phase of the cell cycle. **(A) Cell fractions enriched at specific phases of the cell cycle, were obtained by centrifugal elutriation.** Top panel: representative images of the FACS profiles after PI staining of non-elutriated cells and three different fractions of elutriated material. Bottom graph: cell cycle distribution of each fraction shown as percentage of cells acquired. Error bars = SEM of at least 3 biological replicates. **(B) Expression of the major satellite repeats peaks in G1 phase of the cell cycle whereas minor satellite repeats are expressed in G2/M phase.** The transcript levels of major and minor satellite repeats were analysed by q-RT-PCR in all elutriated fractions of NIH3t3 cells as well as in non–elutriated cells. The relative expression was normalised against *GAPDH* and is shown relative to RNA levels obtained with not elutriated cells. Error bars = SEM of at least 3 biological replicates.
(TIF)

S2 Fig The effect of the proteasome inhibition on the distribution and levels of HP1α. **(A) Delocalisation of HP1α from chromocentres upon proteasome inhibition.** NIH3T3 cells were treated with 20μM MG132 for 8h and immunolabeled with HP1α antibody (green) and co-stained with DAPI (blue). Scale bar 10μm. **(B) Total HP1α protein levels remain similar upon proteasome inhibition**. NIH3T3 cells were treated with 20μM MG132 for 2h, 4h and 8h. Total cell lysate was probed with antibody against HP1α (~22 kDa) and α-tubulin (~55kDa) that served as a loading control.
(TIF)

S1 Table List of primers used throughout this study. (TIF)

## Abbreviations

ActD: Actinomycin
ChIP: Chromatin Immunoprecipitation
HP1: Heterochromatin protein 1
RNA FISH: RNA fluorescent in situ hybridization
RNAPII: RNA polymerase II
MOG: Myelin oligodendrocyte glycoprotein Protein
