# Supplementary figures and images for: Transcriptional Activation of Pericentromeric Satellite Repeats and Disruption of Centromeric Clustering upon Proteasome Inhibition
Source: PLoS One. 2016 Nov 2;11(11):e0165873. doi: 10.1371/journal.pone.0165873 (PMC5091837; doi:10.1371/journal.pone.0165873)

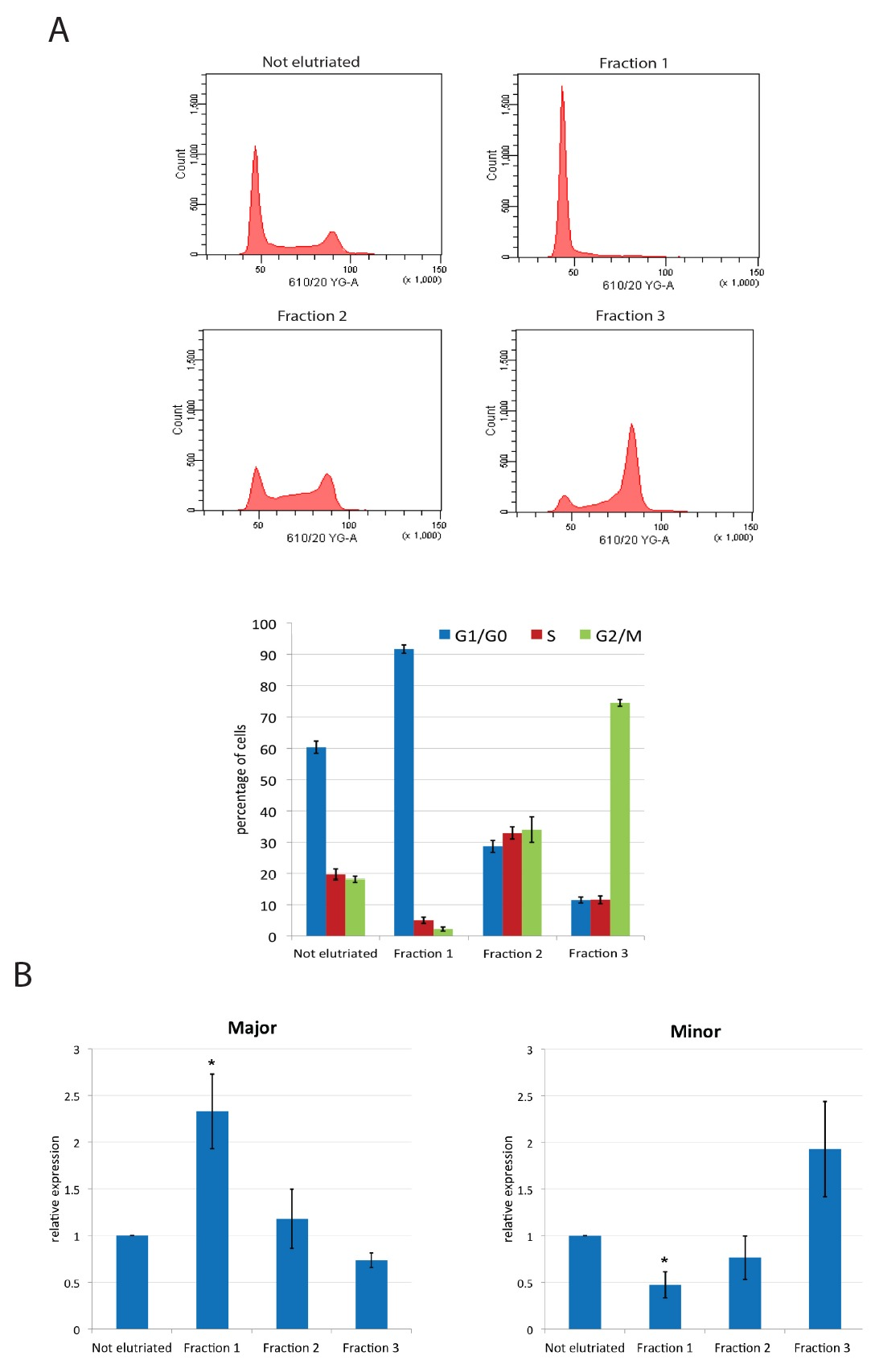

Supplement: S1 Fig — (A) Cell fractions enriched at specific phases of the cell cycle, were obtained by centrifugal elutriation. Top panel: representative images of the FACS profiles after PI staining of non-elutriated cells and three different fractions of elutriated material. Bottom graph: cell cycle distribution of each fraction shown as percentage of cells acquired. Error bars = SEM of at least 3 biological replicates. (B) Expression of the major satellite repeats peaks in G1 phase of the cell cycle whereas minor satellite repeats are expressed in G2/M phase. The transcript levels of major and minor satellite repeats were analysed by q-RT-PCR in all elutriated fractions of NIH3t3 cells as well as in non–elutriated cells. The relative expression was normalised against GAPDH and is shown relative to RNA levels obtained with not elutriated cells. Error bars = SEM of at least 3 biological replicates. (TIF) [file pone.0165873.s001.tif]

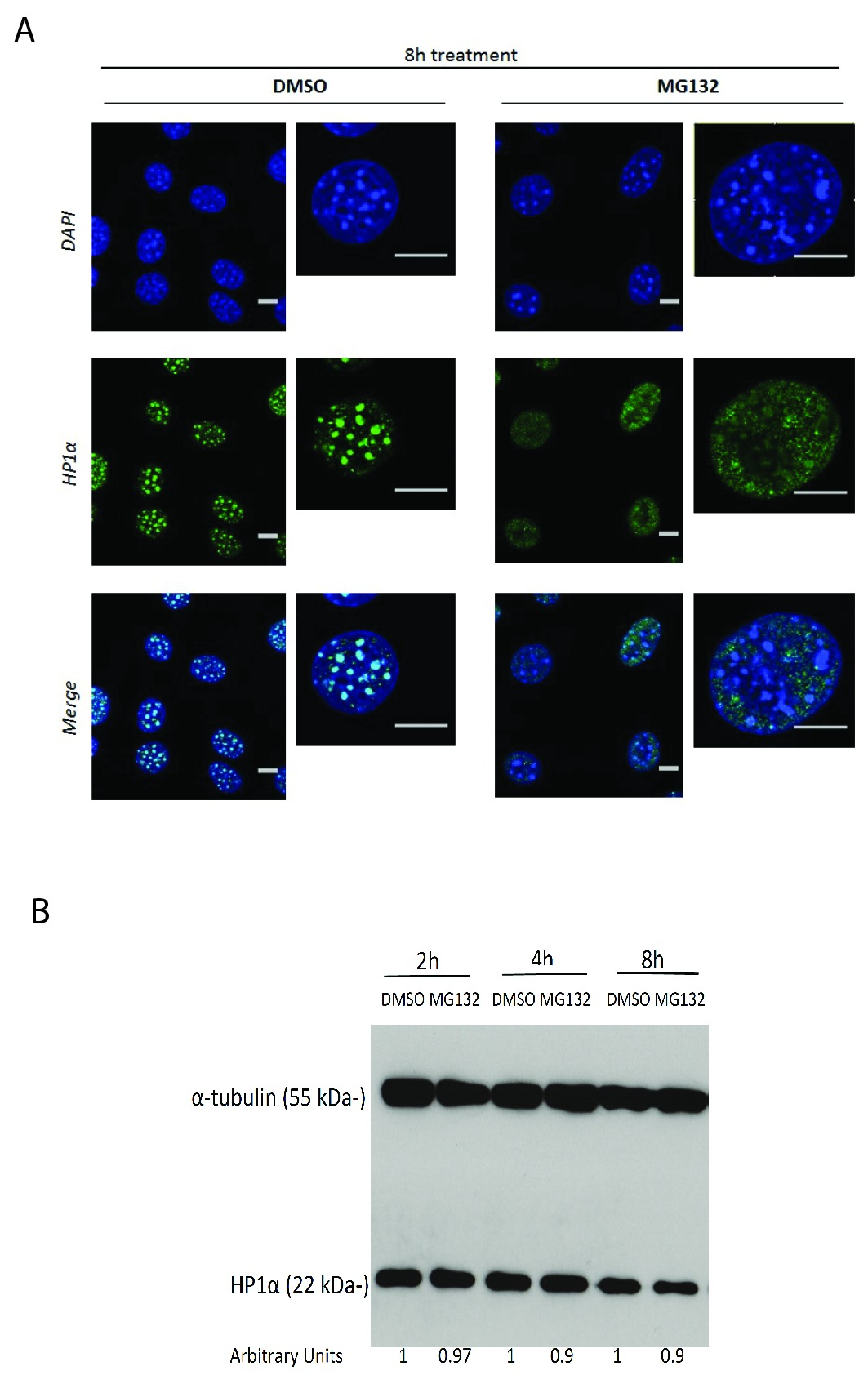

Supplement: S2 Fig — (A) Delocalisation of HP1α from chromocentres upon proteasome inhibition. NIH3T3 cells were treated with 20μM MG132 for 8h and immunolabeled with HP1α antibody (green) and co-stained with DAPI (blue). Scale bar 10μm. (B) Total HP1α protein levels remain similar upon proteasome inhibition. NIH3T3 cells were treated with 20μM MG132 for 2h, 4h and 8h. Total cell lysate was probed with antibody against HP1α (~22 kDa) and α-tubulin (~55kDa) that served as a loading control. (TIF) [file pone.0165873.s002.tif]

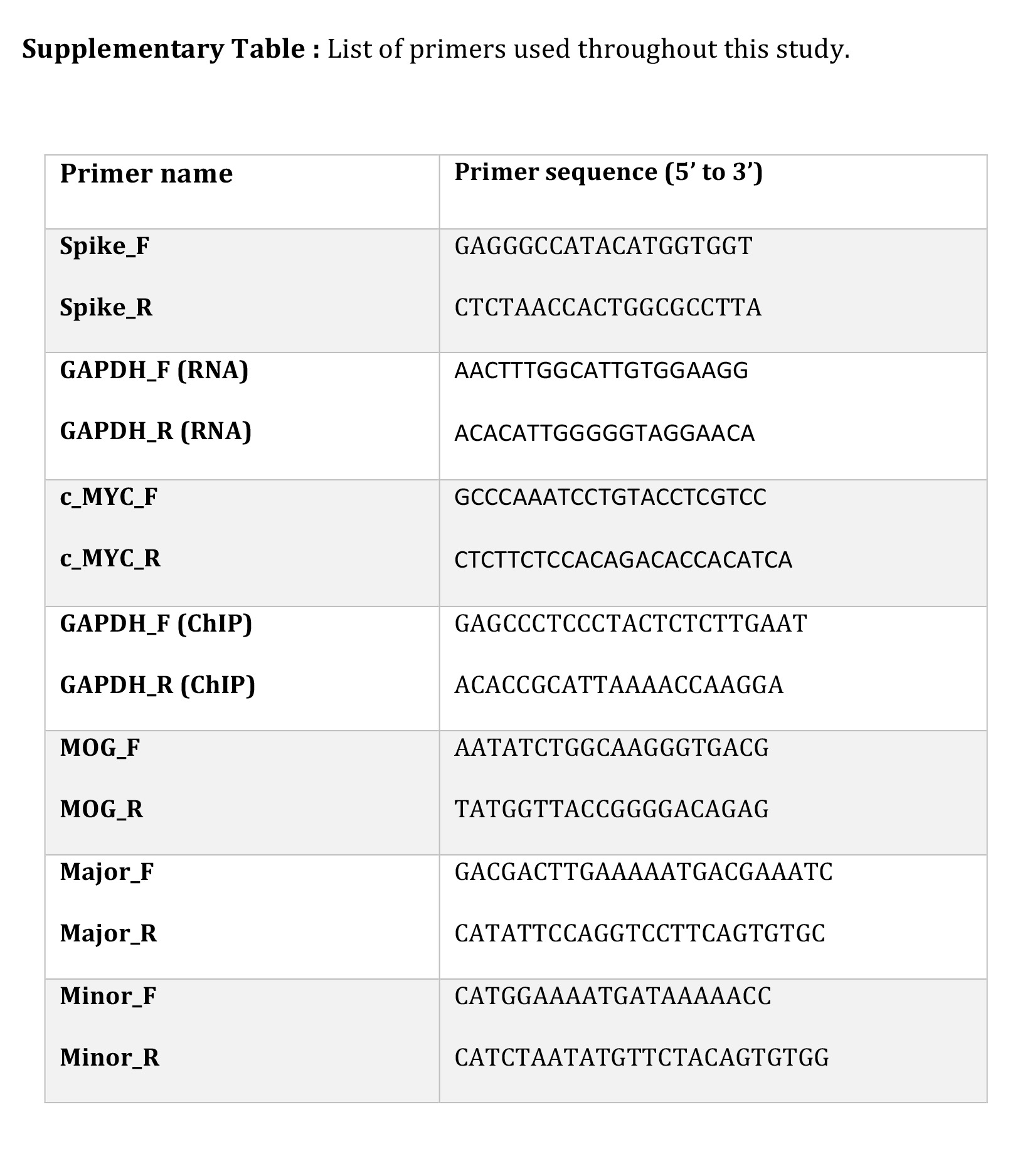

Supplement: S1 Table — (TIF) [file pone.0165873.s003.tif]
